# Visual Working Memory Cannot Trade Quantity for Quality

**DOI:** 10.3389/fpsyg.2018.00719

**Published:** 2018-05-24

**Authors:** Ayelet Ramaty, Roy Luria

**Affiliations:** ^1^The School of Psychological Sciences, Tel Aviv University, Tel Aviv, Israel; ^2^Sagol Department of Neurobiology, University of Haifa, Haifa, Israel; ^3^The Institute of Information Processing and Decision Making, University of Haifa, Haifa, Israel; ^4^Sagol School of Neuroscience, Tel Aviv University, Tel Aviv, Israel

**Keywords:** visual working memory, capacity allocation, resolution, quantity, quality

## Abstract

Two main models have been proposed to describe how visual working memory (WM) allocates its capacity: the slot-model and the continuous resource-model. The purpose of the current study was to test a direct prediction of the resource model suggesting that WM can trade-off between the quantity and quality of the encoded information. Previous research reported equivocal results, with studies that failed to find such a trade-off and other studies that reported a trade-off. Following the design of previous studies, in Experiment 1 we replicated this trade-off, by presenting the memory array for 1200 ms. Experiment 2 failed to observe a trade-off between quantity and quality using a memory array interval of 300 ms (a standard interval for visual WM). Experiment 3 again failed to find this trade-off, when reinstating the 1200 ms memory array interval but adding an articulatory suppression manipulation. We argue that while participants can trade quantity for quality, this pattern depends on verbal encoding and transfer to long-term memory processes that were possible to perform only during the long retention interval. When these processes were eliminated, the trade-off disappeared. Thus, we didn’t find any evidence that the trade-off between quantity for quality can occur within visual WM.

## Introduction

Working memory (WM) enables us to maintain active information about the world, ready to be processed and manipulated according to our goals. WM capacity is correlated with many high cognitive abilities such as academic success (Alloway and Alloway, 2010), top–down attentional control (Kane et al., 2001; Bengson and Mangun, 2011), and fluid intelligence (Vogel et al., 2005; Cowan et al., 2006), corroborating the significant role WM plays in guiding behavior. Previous studies have shown that visual WM has a very limited capacity (Luck and Vogel, 1997; Vogel et al., 2001; Alvarez and Cavanagh, 2004; Awh et al., 2007; Zhang and Luck, 2008), and two major theories have been proposed to explain the nature of visual WM capacity allocation: the *discrete slots* and the *continuous resource*. The discrete slots theory suggests that WM has a fixed number of slots, and each slot could be allocated to represent a certain object. When the number of objects is larger than the number of slots, some objects are left out and will not be stored in WM (Luck and Vogel, 2013). On the other hand, the continuous resource model suggests a more flexible way to divide our resources: namely, it can be distributed between any number of objects. This means that WM is able to store information about all of the objects it is presented with, however, as the number of stored objects increases, each object is represented with a lower resolution (Bays and Husain, 2008).

These two models make different predictions in respect to WM ability to trade quantity (the number of represented items) with quality (the resolution with which these items are represented). The resource model argues that since WM capacity allocation is flexible, it should be possible to represent any given number of items by lowering the resolution of each representation. Thus, visual WM can represent more items with decreased resolution, depending on the task demand and/or instructions. Conversely, according to the slot model a given item could be either represented in WM or it is not represented, depending on whether a slot was allocated (or not) to this item. Thus, once capacity is exceeded, the slot model predicts that trading quality with quantity is impossible. Note that when the number of encoded items is below capacity limits, the slot model can account for such a trade-off (i.e., the slots + averaging account; Zhang and Luck, 2008).

**FIGURE 1:**
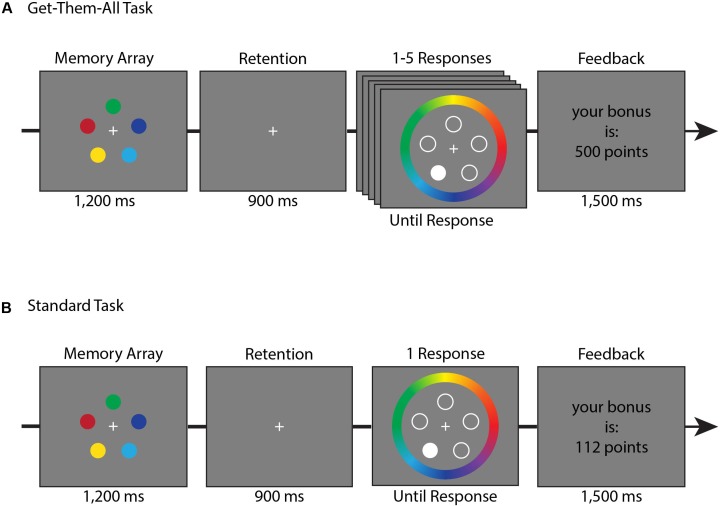
Trial timeline for Experiment 1. **(A)** Get-them-all task trial. **(B)** Standard task trial. The tasks were performed in separate blocks.

Zhang and Luck (2008, based on Wilken and Ma, 2004) developed an analysis method that can separate between the probability (quantity) and the resolution (quality) with which an item is represented in WM. This method is based on analyzing continuous data for colors (or orientations, and even shapes), such that during the test array participants were asked to mark on the color wheel the exact color that was presented in the memory array (see **Figure 1**). Since the color wheel is continuous, it is possible to calculate the deviation between the correct and the reported color (the angle of error). Hence, one can create a histogram of errors. Zhang and Luck argued that this histogram is a convolution of two different distributions – a uniform distribution which is made of random guesses and a von Mises distribution which is made of the remembered colors. Their model separates between two components- Pm and SD. Pm represents the probability that the probe item was stored in memory (depending on the uniform distribution), and SD represents the precision at which that item was represented or the quality of the representation (depending on the width of the von Mises distribution). Using this analysis, it is possible to investigate whether WM can trade-off between quality and quantity and verify the different predictions the slot model and the resource model make.

Zhang and Luck (2011) tried to induce a trade-off between quality and quantity in WM by motivating participants to store more items with lower precision. They conducted three experiments: the first experiment manipulated the amount of precision needed to perform the task by decreasing the number of distinct colors in the color wheel to a small set of nine colors (instead of 180 colors). In the second experiment, participants were given feedback whether their response was correct or incorrect. To encourage encoding more items at the expense of their resolution, Zhang and Luck used a feedback to indicate whether each response was correct, and compared between a low-precision condition which allowed a large range of deviation error for a given response to be considered as correct, to a high-precision condition which allowed only a narrow range of deviation for a correct feedback. In the third experiment, monetary rewards were provided, encouraging participants to reduce precision and to encode more items in WM. Interestingly, all three experiments failed to observe a trade-off between quantity and quality (Bocincova et al., 2016), and Zhang and Luck argued that these results support the slots theory of WM capacity allocation. However, the failure to find a trade-off could also be interpreted as indicating a weak manipulation power. In order to rule out this alternative explanation, Zhang and Luck used a similar manipulation to their Experiment 3 and managed to show a trade-off in iconic memory, indicating that their manipulation was strong enough to produce a trade-off under conditions that required minimal visual WM involvement.

Murray et al. (2012) also conducted a set of experiments to examine whether it is possible to encode more items with lower resolution or fewer items with higher resolution. They manipulated both the expectations about the number of encoded items and the precision required for responding. However, they failed to observe any trade-off between quantity and quality of the information stored in visual WM. In addition, He et al. (2015) manipulated the item similarity between the memory and the test arrays, arguing that high similarity should encourage participants to increase the precision of the encoded items in order to meet the task demands. This manipulation also failed to induce a trade-off between quantity and quality.

In contrast to Zhang and Luck (2011), a recent study by Fougnie et al. (2016), was able to demonstrate a trade-off between quantity and quality in visual WM. Fougnie et al. (2016), contrasted between two tasks – a *standard* task in which participants were asked to report only one color out of five colors that appeared in the memory array, and a *get-them-all* task in which participants were asked to report all five colors. Participants received a monetary reward for both tasks but in different ways, aimed to induce a trade-off. In the standard task, the bonus was directly related to the precision of the response, encouraging participants to respond as accurately as possible. In the *get-them-all* task, the bonus was rewarded only if participants were able to store in memory *all* five presented items, however, the resolution of the stored items didn’t have to be precise (e.g., a response was considered correct if it deviated by less than ±90 degrees from the correct answer). This encouraged participants to store more items with lower resolution. When comparing between the standard and get-them-all task, this study managed to find a significant trade-off between quantity and quality, such that SD and Pm were significantly larger in the get-them-all task compared to the standard task. Namely, participants remembered more items with lower precision in the get-them-all task relative to the standard task. In addition, by providing a cue indicating the number of the items in the memory array, Roggeman et al. (2014) found evidence for a behavioral and a neuronal trade-off in visual-spatial WM.

At first blush, these studies seem to report contradicting results. However, we would like to note one important difference between these studies that according to our view, is responsible for enabling a trade-off between quantity and quality. While Zhang and Luck (2011), Murray et al. (2012), and He et al. (2015) presented the memory array for 200 ms (which is considered a custom interval when measuring visual WM performance), Roggeman et al. (2014) and Fougnie et al. (2016), used a 1,200 and 1000 ms intervals, respectively, notably six or five times longer than the Zhang and Luck study. Several previous studies demonstrated that for simple stimuli such as colors, a 200 ms interval is enough to complete visual WM encoding operations (Alvarez and Cavanagh, 2004; Zhang and Luck, 2011). We argue that this long presentation time is responsible for the trade-off, because it allows other processes besides visual WM encoding and maintenance to take place, such as verbal encoding and transfer to long-term memory (LTM). The goal of the current study is to demonstrate that the trade-off is a result of processes that are not related to visual WM.

We conducted three experiments to support our claim. The first experiment was a direct replication of Fougnie et al. (2016), in which we aimed to reproduce the trade-off between quantity and quality. In two subsequent experiments, we manipulated the ability to verbalize the colors and to use LTM during the memory array interval. Specifically, in Experiment 2, we decreased the encoding duration from 1,200 to 300 ms. A shorter encoding duration discourages the use of verbal encoding and transfer to LTM processes. Experiment 3 used the long encoding duration of 1,200 ms, but included articulatory suppression, thus eliminating verbal encoding and articulatory based transfer to LTM. If indeed the trade-off that was observed by Fougnie et al. (2016) was caused by processes outside visual WM, it should disappear in Experiments 2 and 3.

## Experiment 1: Quantity Quality Trade-Off – Replication

The goal of Experiment 1 was to replicate Fougnie et al. (2016) that found a trade-off between quality and quantity. Since the literature reported several failures to observe such a trade-off (e.g., Zhang and Luck, 2011), and our subsequent experiments failed to find this pattern of result, it was important to show that under some conditions (very long presentation interval and the ability to rehearse the stimuli) the trade-off could be observed.

### Materials and Methods

#### Participants

Twenty naive participants participated in Experiment 1. According to Fougnie et al. (2016, Experiment 1), the effect size was large (Cohen’s *d* between 0.90 and 1.05). Assuming an effect size of 0.9, and alpha of 0.05, to get power of 0.95, 19 participants are needed. Thus, each experiment included twenty participants.

All participants gave informed consent following the procedures of a protocol approved by the Ethics Committee at the Tel Aviv University. The participants were Tel Aviv University students’ who received 40 NIS (approximately $10, plus bonus money, see below) per hour for participation. All participants had normal or corrected-to-normal vision and normal color-vision. This study was approved by the Tel Aviv University ethics committee.

#### Stimuli

Participants were asked to remember the color of five randomly selected colored circles (0.5° radius) that were evenly spaced along an imaginary circle (2.5° radius), centered on a fixation dot. Circle colors were drawn pseudo -randomly (randomly selected, but distinct enough from one another – at least 85 colors away from one another) from a set of 720 equally luminant colors in the CIE L^∗^a^∗^b^∗^ color space (centered at *L* = 54, *a* = 18, *b* = -8, with a radius of 59). Each participant performed two tasks, the *standard task* and the *all get-them-all* *task*. Each task consisted of 3 block of 45 trials.

#### Trial Procedure

The order of the tasks was counter-balanced between participants. The trials began with 1,200 ms presentation of the memory stimulus of five randomly-chosen colored circles, followed by a 900 ms retention interval blank which presented only a white fixation dot in the middle of the screen.

#### Standard Task

Participants’ memory was tested by highlighting one random location (highlighted by presenting a solid white circle at this location while non-probed locations had hollow circles instead), and asking participants to click with the mouse along a circular color wheel (7° radius, centered on fixation) at the exact color they think was presented earlier on that cued-location (**Figure 1A**). After this report, participants were given feedback on how accurate their response was, and were told the amount of bonus points they received (see bonus point section below for more details).

#### Get-Them-All Task

Participants’ memory was tested by asking them to report the color of all five items that were presented in the memory array (**Figure 1B**). While the memory array was identical to the standard task, during the responses phase, participants were asked to report the items sequentially, in a random order. As in the standard task, a random position was highlighted and participants were asked to click with the mouse on the color wheel, where they think was the exact color of the cued-location. If the participant reported a color within ±90° of the correct color, this response was considered correct, and a short feedback was presented. The feedback was a circle that was filled with the correct color while the outer diameter remained the chosen color, providing feedback on the disparity between colors. The feedback was presented for 600 ms. Then the participant was asked about a new item, selected at random from the set of untested positions. If all five items were reported correctly the participant earned bonus points and a sound of cash register “cha-ching” was presented. If one of the responses was incorrect, a sound of buzzer “bzzzz” was presented and the trial ended, awarding zero bonus points.

#### Bonus Points

Participants were motivated to perform well by a monetary reward that was calculated according to their performance in each task (calculated independently for each task). In the standard task, bonus points served to reward participants to minimize the error of the reported item. Participants were given bonus points equal to the inverse error (180 minus the absolute difference in degrees between the response and the true value). In the get-them-all task, 500 bonus points were awarded if the participant got all five items correct. Otherwise zero points were awarded. Participants earned 3.5 NIS (approximately 1$) for every 5,000 points earned. Bonus point rules were explained to participants at the beginning of the experiment. In between trials, text appeared on screen to inform participants of the number of points earned on the previous trial and the cumulative point total for the current task. This feedback was presented for 1,500 ms before a 1,000 ms blank inter-trial interval.

#### Data Analysis

The data from each participant consisted of a set of distances between the original color value and the reported color value in each trial, which reflects the degree of error for each response. We created histograms of these error values to visualize the distribution of responses. We used mixture model analysis (Zhang and Luck, 2008) to decompose our data in each trial into two separate distributions – uniform distribution and von Mises distribution, which are represented by three parameters: Pm (probability of memory), SD (standard deviation), K that represents the number of items the participant stores in memory and μ. Pm represents the probability that the probed item was encoded in memory (in this study we will use guess rate instead of Pm, which is 1-Pm), SD represents the width of the distribution of angles (which are calculated between the correct value and reported value) on trials when the probed item was encoded in memory, which reflects the precision or resolution of the memory representation, and μ represents the center of the von Mises distribution relative to the true value. μ reflects systematic shifts of the distribution away from the original color value. No systematic shifts were observed, thus this parameter will not be further considered. The model was fit separately for each participant and each task. The main reason for testing all responses in the get them all task was to motivate subjects to remember all the items with low resolution, while in the standard task to focus only on a subset of the items but with a higher resolution. In the get-them-all task we analyzed only the first response, following Fougnie et al. (2016). The analysis was made thanks to functions created by Bays et al. (2009). All the data for Experiments 1, 2, and 3 can be found in the Supplementary Material.

### Results and Discussion

**FIGURE 2:**
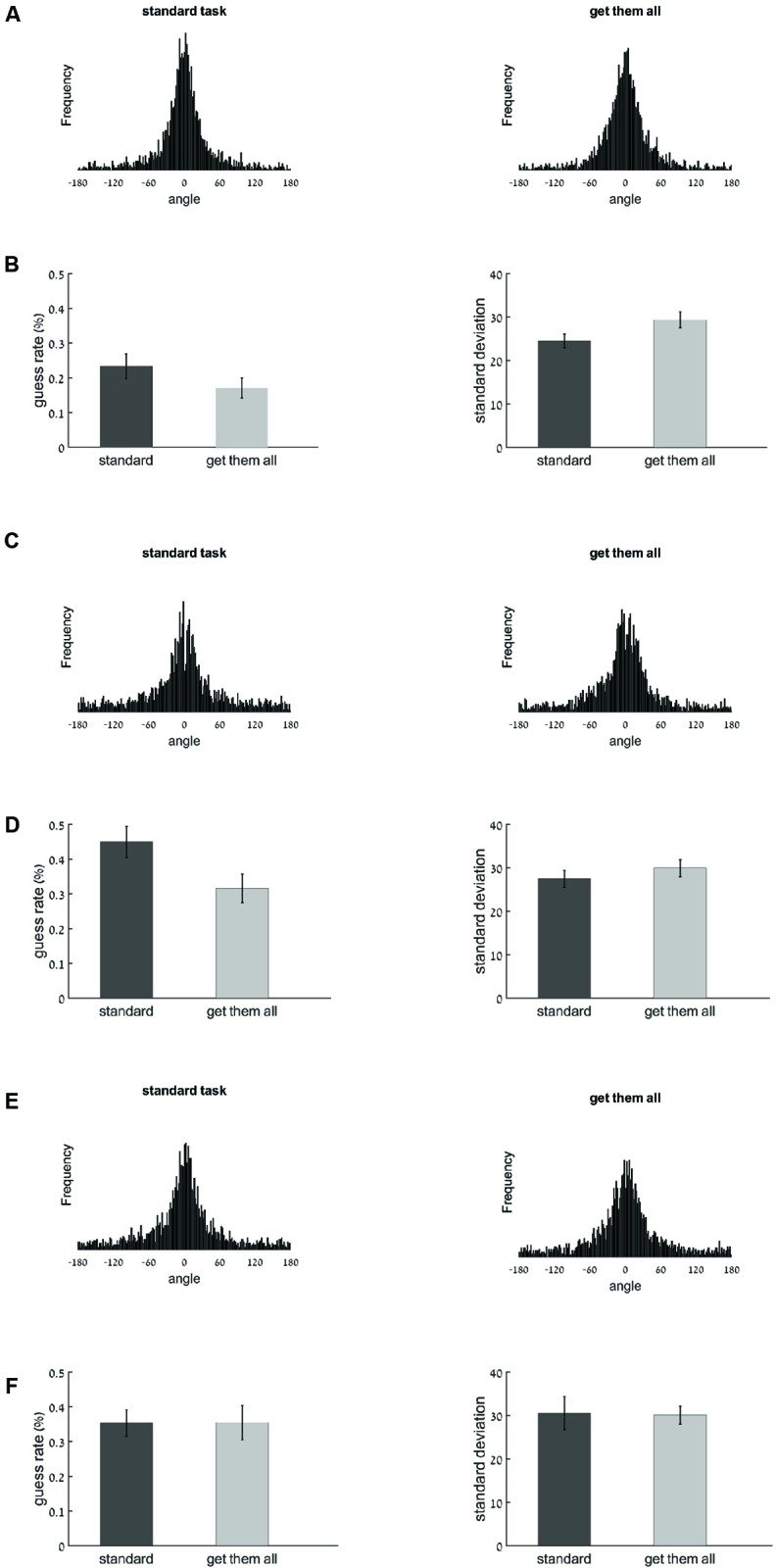
Results of Experiments 1, 2, and 3 (analyzing only the first response in the get-them-all task). In the histograms, the *X*-axis represents the angle between the original color value and the reported color value (the error), the *Y*-axis represents the frequency. **(A)** Histograms displaying error frequency for the standard task (left) and get-them-all task (right) of Experiment 1. **(B)** Model parameter estimates for the Guess rate (1-Pm, on the left) and SD (right) for the standard task (dark gray) and get-them-all task (light gray) of Experiment 1. **(C)** Histograms displaying error frequency for the standard task (left) and get-them-all task (right) of Experiment 2. **(D)** Model parameter estimates for the Guess rate (1-Pm, on the left) and SD (right) for the standard task (dark gray) and get-them-all task (light gray) of Experiment 2. **(E)** Histograms displaying error frequency for the standard task (left) and get-them-all task (right) of Experiment 3. **(F)** Model parameter estimates for the Guess rate (1-Pm, on the left) and SD (right) for the standard task (dark gray) and get-them-all task (light gray) of Experiment 3. Note that larger SD values imply worse precision. Error bars represent *standard error*.

Histograms displaying the degree of error distributions for each task are shown in **Figure 2A**. These distributions were modeled as a mixture of a circular normal and a uniform distribution (Zhang and Luck, 2008) to estimate the quantity (Pm) and quality (SD) of memory. The probability of guessing was significantly lower in the get-them-all task (17%) than in the standard task (23%), *t*(19) = -2.18, *p* < 0.05, and importantly these items were represented with reduced quality: *SD* estimates were larger in the get-them-all task (29.4°) than in the standard task (24.5°), *t*(19) = -2.67, *p* < 0.05^1^ (see **Figure 2B**). The number of items that the participants stored in memory, as represented by K was in the get-them-all task 4.15 and in the standard task: 3.83. These results replicate Fougnie et al. (2016) by showing a trade-off between quantity and quality across the two tasks. We now turn to address the question of what is the underlying mechanism responsible for this trade-off.

## Experiment 2: Trade-Off Disappears When Decreasing the Encoding Duration From 1200 to 300 ms

Important for the present purpose, we note that memory array duration used by Fougnie et al. (2016) and in the current Experiment 1 was 1200 ms, which is a very long retention interval (e.g., six times longer than the duration of the memory array of Zhang and Luck, 2011), who failed to observe such a trade-off. The problem with using such a long encoding interval is that it allows other processes such as verbal encoding and transfer to LTM to affect performance (Vogel et al., 2001; Lin and Luck, 2012). Indeed, it was possible to hear the participants vocally naming the colors while they were doing the task. In the following experiments, we provide direct evidence that the trade-off between quantity and quality disappeared when verbal encoding and the transfer to LTM processes were controlled.

In Experiment 2, we examined whether the trade-off would still occur when participants are given a shorter encoding duration of 300 ms (which is still longer than most of previous WM studies). Note that previous research indicated that 300 ms is ample time to encode simple objects (Vogel et al., 2001; Alvarez and Cavanagh, 2004; Zhang and Luck, 2008; Luria et al., 2010; Lin and Luck, 2012).

If participants are truly able to trade quantity with quality when given sufficient motivation, then decreasing the encoding duration shouldn’t affect this pattern. However, if the trade-off depends on verbal encoding of the memoranda and on LTM processes, rather than on visual WM processes, we predicted that this affect will disappear because a shorter duration limits verbal encoding and transfer to LTM.

### Materials and Methods

Except as noted below all materials and methods were identical to Experiment 1. The only difference is that we used an encoding duration of 300 ms instead of 1,200 ms.

#### Participants

Twenty fresh naive participants participated in Experiment 2. All participants gave informed consent following the procedures of a protocol approved by the Ethics Committee at the Tel Aviv University. The participants were Tel Aviv University students’ who received 40 NIS (approximately $10) per hour for participation. All participants had normal or corrected-to-normal vision and normal color-vision.

### Results and Discussion

Histograms displaying of the degree of error distributions for each task are shown in **Figure 2C**. Interestingly, the quality–quantity trade-off that was observed in the previous experiment disappeared. The difference in SD between the two tasks was no longer significant [27.5° at standard task and 29.9° at get-them-all task, *t*(19) = -1.14, *p* = 0.27] while the difference in guessing rate remained significant (45% at standard task and 32% at get-them-all task), *t*(19) = -5.56, *p* < 0.001, see **Figure 2D**. The number of items that the participants stored in memory, as represented by K was in the get-them-all task 3.4 and in the standard task: 2.75.

This experiment showed that participants still remembered more items at the get-them-all task relative to the standard task, but without significantly trading-off precision. Thus, Experiment 2 provided strong evidence that the trade-off between quantity and quality depended on the long duration interval (1200 ms) used in Experiment 1. Note that previous studies found that increasing the encoding duration had little effect on WM performance for simple objects, such that all WM encoding operations terminate well before 500 ms (Vogel et al., 2001; Alvarez and Cavanagh, 2004; Zhang and Luck, 2008; Luria et al., 2010; Lin and Luck, 2012).

Notably, while a full trade-off was not observed, the difference in guessing rate was still significantly better in the standard task relative to the get-them-all task. The reason may be that an encoding duration of 300 ms is still sufficient for some verbal encoding processes to take place (perhaps for only a subset of the items), but not enough for a refined verbal encoding that can lead to full trade-off between quantity and quality. In Experiment 3, when we directly eliminated verbal encoding by using an articulatory suppression, this difference was no longer significant.

## Experiment 3: Trade-Off Disappears When Using Articulatory Suppression

The goal of Experiment 3 was to directly interfere with the verbal encoding strategy. To this end, we used the same setup as Experiment 1 (presenting the memory array for 1,200 ms), but included an articulatory suppression task. On each trial, two randomly selected digits appeared on the screen and participants were asked to rehearse them out loud. Participants were tested on the digit’s memory at the end of each trial. We reasoned that if the results observed in Experiment 1 depended on using verbal encoding strategies to remember the colors, the trade-off between quantity and quality should disappear once the task includes an articulatory suppression manipulation. Importantly, previous research has demonstrated that an articulatory suppression manipulation did not affect visual WM performance (Luck and Vogel, 1997; Vogel et al., 2001; Todd and Marois, 2004; Luria et al., 2010) while causing a large decrement to verbal memory (Vogel et al., 2001).

### Materials and Methods

Except as noted below all materials and methods were identical to Experiment 1. The only difference was that we included an articulatory suppression task: at the beginning of each trial (both in the standard and get-them-all tasks) two randomly selected digits appeared on the screen. The participants were asked to rehearse these two digits out loud, and at the end of each trial one digit was presented on the screen, and participants had to decide whether this digit appeared at the beginning of the trial or not. The bonus points for each trial in both tasks were rewarded only if they succeeded in this task, otherwise they received zero bonus points.

#### Participants

Twenty fresh naive participants participated in Experiment 3. All participants gave informed consent following the procedures of a protocol approved by the Ethics Committee at the Tel Aviv University. The participants were Tel Aviv University students’ who received 40 NIS (approximately $10) per hour for participation. All participants had normal or corrected-to-normal vision and normal color-vision.

### Results and Discussion

Histograms displaying of the degree of error distributions for each task are shown in **Figure 2E**. Importantly, the quality–quantity trade-off that was observed in Experiment 1, disappeared in Experiment 3 once an articulatory suppression manipulation was included. There was no significant difference between the standard and the get-them-all tasks neither in the guessing rate [35% at standard task and 35% at get-them-all task, *t*(19) = 0.03, *p* = 0.98] nor in the SD [30.5° at standard task and 30.1° at get-them-all task, *t*(19) = 0.11, *p* = 0.91, see **Figure 2F**]. The number of items that the participants stored in memory, as represented by K was in the get-them-all task 3.23 and in the standard task: 3.24.

In this experiment, we used a long encoding duration of 1200 ms similar to Fougnie et al. (2016), however, we directly controlled for verbal encoding by using an articulatory suppression task. Our results clearly show that once the verbal encoding is controlled for, the quantity–quality trade-off disappeared.

## Discussion

This work confirms that under certain circumstances, we can observe a trade-off between the quantity and the quality of the stored information. Experiment 1 demonstrated such a trade-off, by using a long memory array interval of 1,200 ms, replicating Fougnie et al. (2016). Indeed, several additional studies were able to produce such as trade-off. For example, by removing the retention interval, Zhang and Luck (2011) were able to demonstrate a trade-off in iconic memory, using a similar manipulation that failed to produce a trade-off in visual WM, Eriksen and Yeh (1985) observed a trade-off in spatial resolution when selecting items (cf., Franconeri et al., 2007) and Roggeman et al. (2014) observed a trade-off in spatial WM (albeit with a memory array of 1000 ms).

These studies indicate that a trade-off between quantity and quality is possible for processes and mechanisms that are ‘outside’ the visual WM workspace.

We argue that using a very long presentation interval (Roggeman et al., 2014; Fougnie et al., 2016) enabled processes such as verbal encoding and transfer to LTM, and that these processes, and not visual WM, are responsible for the trade-off. Indeed, Experiments 2 and 3 eliminated the trade-off between quantity and quality by using a memory array of 300 ms, or by adding an articulatory suppression manipulation before the 1,200 ms memory array interval. Several previous studies have shown that visual WM is not affected by an articulatory suppression manipulation (Luck and Vogel, 1997; Vogel et al., 2001; Todd and Marois, 2004; Luria et al., 2010) or by increasing the memory array interval up to 500 ms (Vogel et al., 2001; Alvarez and Cavanagh, 2004; Zhang and Luck, 2008; Luria et al., 2010; Lin and Luck, 2012). Thus, our manipulations did not affect visual WM encoding or maintenance processing.

One option is that verbal encoding allowed participants to remember the colors as labels (“blue,” cf. Bae et al., 2015) and then use the long retention interval to transfer this categorical verbal information into LTM (e.g., Carlisle et al., 2011). This process will likely produce a trade-off because it allows for more categorical (and hence imprecise) information to be stored in LTM. Once we eliminated this possibility, either by reducing the retention interval or by using articulatory suppression manipulation, the trade-off disappeared.

Using a long retention interval may allow more time for eye movements and fixations on each encoded item. Thus, one option is that fixating the targets increase their encoded resolution. However, in Experiment 3, we also used a long retention interval that allowed sufficient time for target fixation, but we did not observe any change in the color resolution when controlling for verbal encoding.

Note that the long presentation interval by itself, only allowed participants to engage in other processes that induced the trade-off. Thus, we do not argue that visual WM cannot be measured with long memory array intervals. For example, a recent study (Souza and Skóra, 2017) have demonstrated that when given enough time (by using serial presentation) verbal encoding of colors can increase both the quality and quantity of the stored information.

Another key element in producing a trade-off, is that participants were highly motivated to encode more items with a low resolution. However, we argue that participants were equally motivated in Experiment 2 and in Zhang and Luck (2008) study, but lacked the possibility to trigger verbal encoding and transfer to LTM processes.

Our approach to eliminate such processes was akin to a study by Lin and Luck (2012), who investigated why prior research (Hartshorne, 2008; Makovski et al., 2008) found that LTM traces affected WM performance. Lin and Luck noted that prior research used a long retention interval of 1 s, and by reducing this interval to 200 ms, they eliminated the LTM effects.

Research investigating whether visual WM can trade quantity for quality was motivated by the debate between the flexible resource model and the slot model. While the resource model can naturally explain any flexible allocation of WM capacity, it was argued that the slot model has limited flexibility because slot are an all-or-none mechanism. While the slot model specifically argues that if a slot was not allocated to an item, this item is not encoded in WM workspace, the slot model still has a great deal of flexibility. First, when the encoded array is below capacity limits, it is possible to allocated more than one slot to a given item (Zhang and Luck, 2008), which means that it can explain a trade-off between quantity and quality in this situation. Second, the slots model can explain how WM can encode only a specific feature of an item (Woodman and Vogel, 2008; Luria et al., 2010; Cowan et al., 2013). Thus, although the slot model does not predict a trade-off under the current experimental conditions (because the represented memoranda exceeds WM capacity limitations), it does incorporate several flexible mechanisms.

To summarize, the current experiments found a trade-off between quantity and quality but corroborating previous studies (Zhang and Luck, 2011), we attribute this effect to verbal encoding and transfer to LTM processes, rather than to visual WM.

## Ethics Statement

This study was carried out in accordance with the recommendations of Ethics Committee at the Tel Aviv University with written informed consent from all subjects. All subjects gave written informed consent in accordance with the Declaration of Helsinki. The protocol was approved by the Tel Aviv University ethics committee.

## Author Contributions

AR conducted this research under the supervision of RL which is the head of the laboratory.

## Conflict of Interest Statement

The authors declare that the research was conducted in the absence of any commercial or financial relationships that could be construed as a potential conflict of interest.

## Footnotes

1 After the experiment was completed, we asked participants how they remembered the colors. They reported that in the get-them-all task they used verbal categorical representations in order to better remember the colors.

## Supplementary Material

TABLE S1 Results for Experiment 1 (replication).

TABLE S2 Results for Experiment 2 (300 ms).

TABLE S3 Results for Experiment 3 (articular suppression).
